# Preparation, Characterization, and Performance Evaluation of Polysulfone Hollow Fiber Membrane with PEBAX or PDMS Coating for Oxygen Enhancement Process

**DOI:** 10.3390/polym10020126

**Published:** 2018-01-28

**Authors:** Kok Chung Chong, Soon Onn Lai, Woei Jye Lau, Hui San Thiam, Ahmad Fauzi Ismail, Rosyiela Azwa Roslan

**Affiliations:** 1 Lee Kong Chian Faculty of Engineering and Science, Universiti Tunku Abdul Rahman, Jalan Sungai Long, Bandar Sungai Long, Kajang 43300, Malaysia; laiso@utar.edu.my (S.O.L.); thiamhs@utar.edu.my (H.S.T.); 2 Advanced Membrane Technology Research Centre (AMTEC), Universiti Teknologi Malaysia, Skudai 81310, Johor, Malaysia; afauzi@utm.my (A.F.I.); rosyielaazwa.roslan@yahoo.com (R.A.R.)

**Keywords:** hollow fiber membrane, oxygen enrichment, polysulfone, PEBAX, PDMS

## Abstract

Air pollution is a widely discussed topic amongst the academic and industrial spheres as it can bring adverse effects to human health and economic loss. As humans spend most of their time at the office and at home, good indoor air quality with enriched oxygen concentration is particularly important. In this study, polysulfone (PSF) hollow fiber membranes fabricated by dry-jet wet phase inversion method were coated by a layer of polydimethylsiloxane (PDMS) or poly(ether block amide) (PEBAX) at different concentrations and used to evaluate their performance in gas separation for oxygen enrichment. The surface-coated membranes were characterized using SEM and EDX to determine the coating layer thickness and surface chemical properties, respectively. Results from the gas permeation study revealed that the PSF membrane coated with PDMS offered higher permeance and selectivity compared to the membrane coated with PEBAX. The best performing PDMS-coated membrane demonstrated oxygen and nitrogen gas permeance of 18.31 and 4.01 GPU, respectively with oxygen/nitrogen selectivity of 4.56. Meanwhile, the PEBAX-coated membrane only showed 12.23 and 3.11 GPU for oxygen and nitrogen gas, respectively with a selectivity of 3.94. It can be concluded the PDMS coating is more promising for PSF hollow fiber membrane compared to the PEBAX coating for the oxygen enrichment process.

## 1. Introduction

Air pollution is a serious environmental issue that is widely discussed around the globe, especially in the developing countries. The article published by the Lancet Respiratory Medicine in 2012 reported that 12.6 million deaths recorded worldwide were related to environmental pollution and, out of that number, 8.2 million were caused by air pollution [1]. The study had also shown that indoor air pollution was the major factor contributing to the adverse health problems of humans [1]. A poor level of indoor air quality is likely to cause sick building syndrome that affects both productivity and personal health. One of the effective ways to improve indoor air quality is to supply oxygen-enriched air to enhance air freshness [2]. Additionally, oxygen-enriched air is highly sought after in medical applications for hypoxemic patients and internal combustion engines to reduce the environment pollution that arises from unburned hydrocarbons [3,4].

Currently, two conventional techniques that are commonly used in oxygen gas production are pressure swing absorption (PSA) and cryogenic distillation. These two techniques are commercially utilized in industrial applications to produce large volumes of high purity gases. Membrane technology, on other hand, is an emerging technology which is able to produce gases in relatively good purity at a moderate production volume. The main advantages of membrane technology compared to conventional methods are that membrane technology requires less setting up space and is less energy intensive [5]. 

Generally, membranes act as a barrier for the gas separation whereby its characteristics and properties play an important role in the separation performance [6]. Nevertheless, the gas separation performance is always limited by the selectivity and permeability trade-off as shown in the Robeson upper bound [7,8]. Permeability describes the ability of membrane to allow gas molecules to diffuse through the matrix and is governed by the Fick’s diffusion law [9]. Selectivity meanwhile describes the ratio of the permeance of species A with respect to species B. To date, most of the works carried out are focused on the development of advanced polymeric-based membranes that demonstrate superior permeability and selectivity in lab-scale applications [10]. 

One of the easiest and simplest ways to improve the existing performance of polymeric-based membranes is via surface coating. Polydimethylsiloxane (PDMS) and poly(ether block amide) (PEBAX) are widely used as coating materials for membrane to minimize not only its surface defects but also improve its separation performance. Studies showed that PDMS coating is able to repair membrane surface defects by eliminating the non-selective membrane pores and to enhance gas pair selectivity [11,12]. Wahab et al. [13] and Moaddeb and Koros [14] reported the potential use of PDMS-coated membranes to improve the gas separation process, especially in CO_2_ separation for combustion. Unlike PDMS, PEBAX contains multiblock copolymers which consist of rigid polyamide chains separated by flexible polyether fragments. Previous work showed that the good species separation performance of PEBAX-coated membrane is attributed to its soft block of polyether fragments that allows gas molecules to permeate easily [15,16]. The hard polyamide block meanwhile provides good mechanical strength for the membrane [16]. Because of this, PEBAX was applied as a coating material on the membrane to evaluate its performance in gas separation. Kim et al. [15] reported that PEBAX-coated polyacrylonitrile (PAN) membrane was able to improve the sour gases (SO_2_ and CO_2_) separation performance from nitrogen. Liu et al. [16] on the other hand found that polysulfone (PSF) flat sheet membranes coated with PEBAX via dip coating method were able to improve the permselectivity of CO_2_/N_2_. 

In this study, two different coating materials i.e., PDMS and PEBAX were used to improve the separation properties of PSF hollow fiber membranes particularly for oxygen/nitrogen separation. The outer surfaces of membranes were coated with polymers at different concentrations followed by instrumental characterization before proceeding to gas separation performance evaluation. 

## 2. Materials and Methods 

### 2.1. Materials

The main material used for hollow fiber membrane fabrication was commercial PSF with the trade name UDEL-3500 (Amocco Chemicals, GA, USA). The solvent (*N*,*N*-dimethylacetamide, DMAc) and co-solvent (ethanol, EtOH) used to dissolve PSF pellets were obtained from Merck (Darmstadt, Germany), Tetrahydrofuran (THF) with purity >99% purchased from QReC (Selangor, Malaysia) was also used as co-solvent during dope solution preparation. The PDMS and PEBAX coating materials were purchased from Sigma-Aldrich, St. Louis, MO, USA and Arkema, PA, USA, respectively. They were used to form a selective layer on the outer surface of hollow fiber membrane. *N*-hexane obtained from Merck (Darmstadt, Germany) was used to prepare PDMS coating solution.

### 2.2. Fabrication of Hollow Fiber Membrane

The dope solution employed for the hollow fiber membrane fabrication was composed of 30 wt % PSF, 30 wt % DMAc, 30 wt % EtOH, and 10 wt % THF. Before dissolving in the solvent, the PSF pellets were dried in vacuum oven for one day at 70 °C to completely remove the moisture. The PSF pellets were then slowly added into the solution containing DMAc, EtOH, and THF under mechanical stirring. After the PSF pellets were completely dissolved, the mixture was continuously stirred for another 24 h in order to obtain a homogenous solution. The dope solution was then degassed in ultrasonic bath for 4 h to remove the air bubbles trapped in the solution. 

**Figure 1:**
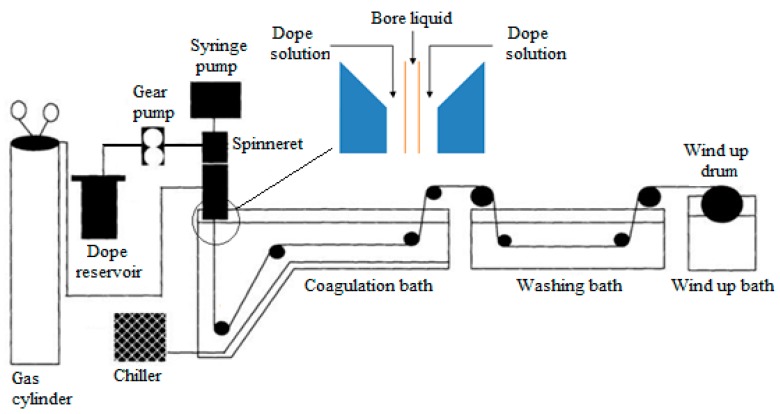
Schematic diagram of the dry-jet wet phase inversion apparatus setup for PSF hollow fiber membrane fabrication [2].

Dry-jet wet phase inversion method was employed in this study to fabricate the PSF hollow fiber membrane and detailed description of the method can be found elsewhere [17,18]. The spinning machine is schematically shown in Figure 1 and the detailed spinning parameters are shown in Table 1. The dope solution was first transferred to a stainless-steel dope reservoir prior to the fabrication process. A gear pump was used to deliver the dope solution from the reservoir to the spinneret, passing through the annular spinneret. Bore fluid composed of distilled water would flow through the center of the spinneret to instigate the solvent/non-solvent exchange between the dope solution and bore fluid, forming lumen as shown in the insertion in Figure 1.

Nascent hollow fiber membrane was formed when it was contacted with large amount of water in the coagulation bath. The hollow fiber was then guided manually to the washing bath before being collected by wind-up drum. The hollow fibers collected from the drum were subjected to two-day immersion in water bath in order to completely remove the solvent residual. Lastly, the hollow fiber membranes were dried in the vacuum oven at 70 °C for one day in order to remove the moisture/residual solvent content. As the solvents from the dope solution are considered hazardous and will eventually be discharged into the drain, post-treatment using solvent absorbents is recommended. However, as our work only dealt with lab-scale membrane fabrication, there is no such treatment process implemented for the time being [19]. For large scale membrane fabrication process by phase inversion method, wastewater treatment process is required in order to treat the effluent containing solvents before being discharged to environment [20].

### 2.3. Coating of Membrane Surface

In this study, the coating solutions containing either PDMS or PEBAX were prepared at three different concentrations, i.e., 1, 3, and 5 wt % and were used to form a thin coating layer on the outer surface of hollow fiber membrane. To prepare the PDMS coating solution, the PDMS base was first mixed with the predetermined volume of n-hexane solution to obtain the desired weight percentage. The solution was continuously stirred for 2 h followed by 4 h sonication to remove microbubbles trapped in the solution. The dried PSF membranes were then dipped in the PDMS coating solution for 2 min before subjecting to heat treatment (curing step) in an oven at 70 °C for 4 h. The procedures were repeated five times in order to obtain good PDMS coating layer. 

To prepare the PEBAX coating solution, PEBAX with specific quantity was first dissolved in a solution composed of distilled water and ethanol at volume ratio of 30:70. The mixture was then stirred at room temperature for 2 h prior before proceeding to the sonication process for 4 h. Similar to PDMS coating technique, the hollow fiber membranes were dipped into the PEBAX solution for 2 min followed by drying at 70 °C for 4 h. Similar to the PDMS coating protocol, the procedures were repeated five times in order to obtain good PEBAX coating layer. It must be noted that during the curing process, the PEBAX-coated fibers had to be kept separately to avoid them from sticking. Table 2 shows the types of the hollow fiber membranes coated with different materials at different concentrations.

### 2.4. Membrane Characterization

The cross sectional and morphology of the hollow fiber membrane were carefully examined by scanning electron microscope (Hitachi, Tokyo, Japan, S3400N). Prior to analysis, the hollow fiber membranes were cryogenically cracked using liquid nitrogen in order to obtain a clear morphology of the membrane without defect. The samples were then coated with a layer of gold using sputter coating machine (Emitech, East Sussex, UK, SC7620) to improve the surface conductivity [21]. Energy dispersive X-ray (EDX) spectrometer was used to analyze the elements present on the membrane outer surface. The element that can be used to confirm the existence of the coating layer were silicon (Si) for PDMS coating and nitrogen (N) for PEBAX coating. For each membrane sample, the membrane characterization was repeated five times to yield the average results with standard deviation.

### 2.5. Gas Permeation Study

**Figure 2:**
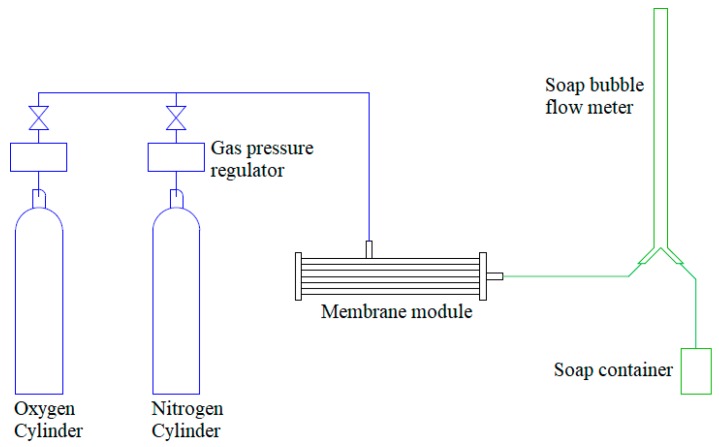
Schematic diagram of gas permeation study [2].

The gas permeation setup is schematically illustrated in Figure 2. In this study, the gases used for experiment were pure oxygen and nitrogen gas supplied by MegaMount Industrial Gas, Johor, Malaysia with purity >99.99%. Five units of hollow fiber membranes with the length of 23 cm were bundled and placed within the stainless steel membrane housing. The hollow fiber membranes were sealed with dead end manner using epoxy resin (Loctite, Düsseldorf, Germany). The flow arrangement in this study was shell-side feed where the feed gas from the cylinder would diffuse through the outer surface of the hollow fiber membranes and the permeated gas will pass through the lumen of fibers. The permeated gas is connected to a soap bubble flow meter in which the gas permeance could be determined as

(1) $$\frac{P_{A}}{l}=\frac{273.15\times 10^{6}Q}{A\Delta PT}$$

where *P_A_*/*l* is the gas permeance (GPU) (Note: 1 GPU = 10^−6^ cm^3^ (STP)/cm^2^ cm Hg), *Q* is the volumetric flowrate of gas diffuse across the membrane (cm^3^/s, STP), *A* is the effective membrane area (cm^2^), Δ*P* is the transmembrane pressure (cm Hg), and *T* is the temperature. The experiments were carried out at room temperature (28 °C) with constant feed pressure of 5 bar. For each membrane sample, the experiments were repeated five times to yield the average results with standard deviation. The membrane selectivity, *α_A_*_/*B*_ describing the ratio of gas pair permeability can be calculated by the pressure normalized flux ratio of oxygen over nitrogen gas.

(2) $$\alpha_{A/B}=\frac{P_{O2}}{P_{N2}}$$

## 3. Results and Discussion

### 3.1. Membrane Morphology

**Figure 3:**
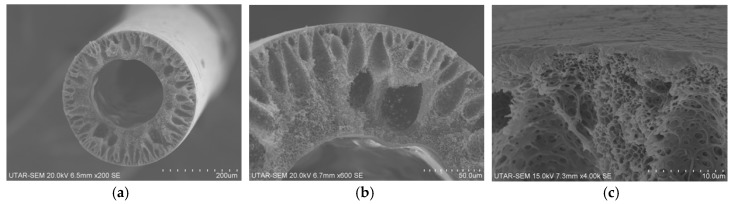
SEM image of uncoated PSF membrane: (**a**) overall cross section; (**b**) partial cross section; and (**c**) top skin layer.

The SEM images for the uncoated PSF membranes fabricated via dry-jet wet phase inversion method are shown in Figure 3. As can be seen, a thin and dense structure can be found at both inner and outer layer of the membrane. Teardrop-like structure is found to dominate the cross-section morphology of the membrane. In addition, spongy-like structure is seen at the section close to the inner part of the membrane. The formation of thin dense structure at the inner and outer layer of the membrane is likely due to the use of high concentration of polymer dope solution (30 wt % PSF) that slows down the solvent/non-solvent exchange due to increased dope viscosity. The formation of the teardrop-like structure meanwhile can be attributed to the use of strong non-solvent (water) in the bore fluid and coagulation bath that promotes aggressive exchange between the solvents in the dope solution and the water [22,23,24]. 

**Figure 4:**
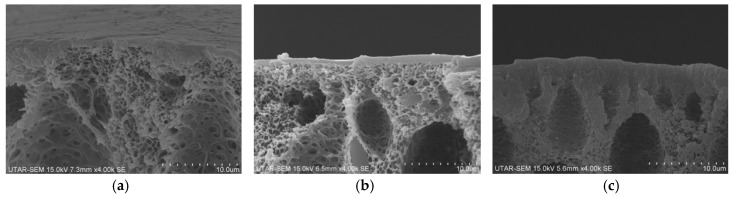
SEM image of top surface of PSF membrane: (**a**) uncoated membrane; (**b**) 1 wt % PDMS coating; and (**c**) 1 wt % PEBAX coating.

Figure 4 compares the top surface of PSF hollow fiber membranes coated with PDMS and PEBAX with the uncoated membrane. One can observe that additional layer is formed on the top surface of PDMS- and PEBAX-coated membranes upon coating. With increasing the coating solution concentration from 1 to 5 wt %, the coating layer thickness of PDMS- and PEBAX-coated membranes increases from 0.7 to 1.7 μm and from 0.3 to 1.2 μm, respectively, as shown in Table 3. Meanwhile, Figures S1 and S2 shows the SEM images of membrane top surface coated with concentrations of PDMS and PEBAX solution. Our findings are in good agreement with the previous studies in which increasing the concentration of coating solution increased the coating layer thickness of membranes [25,26].

### 3.2. Effects of PDMS and PEBAX Coating on Membrane Surface Chemistry

Table 4 shows the EDX results on the surface of PSF hollow fiber membranes coated with different conditions. The area selected for each sample’s surface survey is shown in Figure S3. The detection of carbon, oxygen, and sulphur on the membrane surface can be attributed to the characteristics of the PSF itself that contains subunit of aryl-SO_2_-aryl. Upon coating with different polymeric materials at various concentrations, the detection of new element corresponding to the coating material can be found. For the PDMS coating, the detection of silicon on the membrane surface is the best indication on the presence of PDMS layer on the membrane surface and the increase in the PDMS coating solution results in higher amount of silicon detected. Similarly, nitrogen corresponding to the PEBAX can only be found in the membranes coated with the PEBAX. As the coating layer becomes thicker with the use of higher concentration of PEBAX solution, higher amounts of nitrogen are found. The increase in the amount of element detected (silicon for the PDMS-coated membrane and nitrogen for the PEBAX-coated membrane) is in good agreement with the increase in the respective coating layer thickness. Nevertheless, it is interesting to note that the thickness for the PEBAX coating is relatively lower than that of PDMS coating at the same concentration. According to Wang et al. [25] and Espositio et al. [26], PEBAX solution is less viscous and has ability to penetrate to the membrane surface pore more easily and form rapid gelation during curing process.

### 3.3. Effects of Coating on Membrane Performance

**Figure 5:**
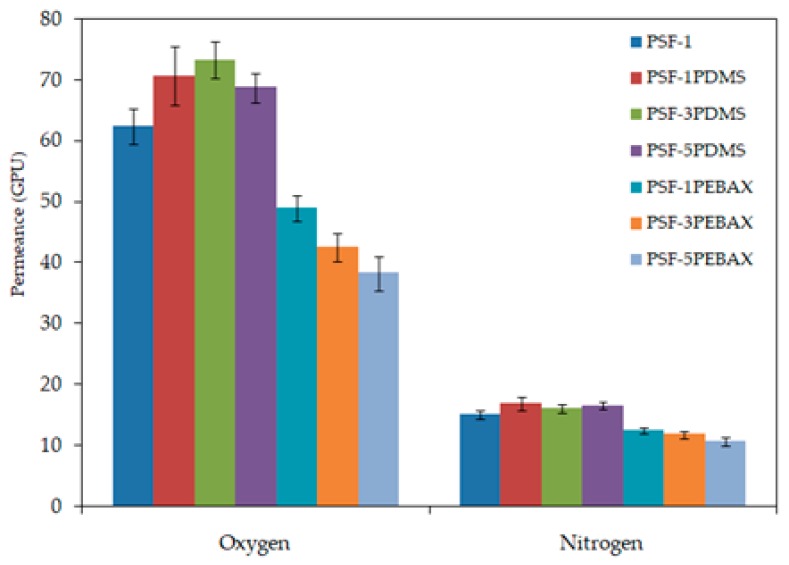
Effect of PDMS and PEBAX coating on the PSF membrane with respect to gas permeance.

The effect of surface coating on the hollow fiber membrane performance with respect to gas permeation and selectivity were evaluated and the results are shown in Figure 5 and Table 5, respectively. The gas permeances of the membranes are in the range of 38–73 GPU for oxygen gas and 10–17 GPU for nitrogen gas. The uncoated membrane shows 62.35 and 15.11 GPU for the oxygen and nitrogen gas, respectively. As a comparison, the PDMS-coated membranes generally show higher gas permeance performance for both oxygen and nitrogen gas. The membrane coated with 1 and 3 wt % PDMS solution demonstrates 70.64 and 73.25 GPU, respectively for oxygen gas and 16.82 and 16.05 GPU, respectively for nitrogen gas.

The membrane coated with optimum concentration of PDMS solution (i.e., PSF-3PDMS) in particular exhibits 17.5% and 11.3% enhancement for oxygen and nitrogen gas permeance, respectively, in comparison to the results shown by the uncoated membrane (i.e., PSF). Results show that the excessive use of PDMS solution could negatively affect the membrane gas permeance. It is because the PSF-5PDMS membrane shows lower oxygen gas permeance than the PSF-1PDMS and PSF-3PDMS membranes. When the membranes are coated with low concentration of PDMS solution, the high affinity of PDMS towards oxygen molecules tends to improve the oxygen gas permeance of membrane without affecting the permeance of nitrogen gas. Coating the membrane surface with highest PDMS concentration however reduces the gas permeance because of increased diffusivity of resistance caused by the thicker PDMS layers formed. Other works have also reported the reduction in gas permeance following the use of high concentration of PDMS coating solution [27,28].

Meanwhile, it is found that the PEBAX-coated membranes in general show lower gas permeance for oxygen and nitrogen than that of uncoated membrane. Our findings are opposite compared to the works carried out by Wahab and Sunarti [29] and Wang et al. [25] in which the membrane gas permeance increased upon PEBAX coating on the flat sheet polymeric membranes. One of the main reasons contributing to the inconsistent findings is the use of membrane in hollow fiber configuration. The PEBAX coating procedure that is normally adopted for flat sheet membrane surface modification seems not effective to form good integrity coating layers on the hollow fiber surface. As PEBAX coating solution (on membrane surface) could experience rapid gelation during the drying process, it makes the fibers sticking together and/or being stuck with drying platform. This, as a result, affects the integrity of coating layer and the membrane performance. In order to tackle the issue, optimization on the PEBAX coating conditions particularly for hollow fiber membrane (circular shape) is required and will be reported in our coming research work.

### 3.4. Comparison of Oxygen and Nitrogen Gas Separation with Previous Studies

The extensive literature search reveals that many membrane gas separation studies focused on the carbon dioxide related gas separation such as CO_2_/N_2_, CO_2_/O_2_, and CO_2_/CH_4_ [30,31]. To the best of our knowledge, there are very few studies reporting the performance of hollow fiber membranes for oxygen/nitrogen separation. Table 6 summarizes the relevant works that have been previously carried out on the oxygen/nitrogen gas separation using either mixed matrix PSF membranes or membranes coated with PDMS or PEBAX. As can be seen, besides demonstrating higher permeance for oxygen and nitrogen gas, the surface-coated PSF membranes used in this study could still maintain reasonably high gas pair selectivity compared to most of the studies. The enhanced performance can be attributed to the unique structure of the hollow fiber membrane that possesses teardrop-like structure in the membrane cross-section, leading to lower mass transfer resistance for gas molecules. Although the work done by Prajapati et al. [32] showed that the gas pair selectivity of the hollow fiber membrane could be further increased with increasing PDMS concentration from 5 to 20 wt %, significant reduction in permeance was reported. Thus, they suggested that the coating solution concentration should be controlled at less than 5 wt %. Prajapati et al. [32] also suggested to reduce the concentration of toxic solvent (DMAc) during dope solution preparation. It is because by there was little effect on the membrane gas separation performance by reducing the DMAc concentration from 39 to 30 wt %. In order to further improve the coating layer properties of the hollow fiber membrane for gas separation, optimizing the coating solution and its conditions (both fabrication and post-treatment) are required.

## 4. Conclusions

In this study, the surface properties of the PSF hollow fiber membranes were modified by subjecting the membranes to dip-coating process using either PDMS or PEBAX at different concentrations. Results showed that the membranes coated with PDMS exhibited better permeance and selectivity in oxygen/nitrogen separation process in comparison to the membranes coated with PEBAX. Upon coating with 3 wt % PDMS, the membrane exhibited the best performance, showing permeance of 18.31 and 4.01 GPU for oxygen and nitrogen gas, respectively and recording oxygen/nitrogen selectivity of 4.56. Meanwhile, the best performing PEBAX-coated membrane (1 wt % PEBAX) only showed 12.23 and 3.11 GPU for oxygen and nitrogen gas, respectively and selectivity of 3.94. Although previous research works have shown that the PEBAX coating layer is more selective compared to the PDMS coating layer, contradictory results were obtained in this work. This is likely because the previous works only investigated the effects of PEBAX coating on the flat sheet membranes and when the same conditions were applied to the circular surface of hollow fiber membrane, it led to different results. As a conclusion, PDMS coating is more promising to improve the performance of PSF hollow fiber membrane for oxygen enrichment process compared to the PEBAX coating. 

## Figures and Tables

**Table 1:** Spinning parameter of PSF hollow fiber membrane.

Spinning Parameter	Value
Spinneret OD/ID (mm/mm)	0.6/0.3
Bore liquid	Distilled water
Bore liquid temperature (°C)	25
Bore liquid flow rate (mL/min)	0.3
External coagulant	Tap water
External coagulant temperature (°C)	25
Air gap (cm)	30
Room relative humidity (%)	55 ± 5

**Table 2:** Coating conditions for PSF-based hollow fiber membranes.

Membrane ^a^	PDMS Coating (wt %)	PEBAX Coating (wt %)
PSF	–	–
PSF-1PDMS	1	–
PSF-3PDMS	3	–
PSF-5PDMS	5	–
PSF-1PEBAX	–	1
PSF-3PEBAX	–	3
PSF-5PEBAX	–	5

^a^ The hollow fiber PSF membrane used in this study was composed of 30 wt % PSF, 30 wt % DMAc, 30 wt % EtOH, and 10 wt % THF.

**Table 3:** Coating thickness for the as spun hollow fiber membranes

Membrane	Thickness (μm)
PSF-1PDMS	0.7 ± 0.07
PSF-3PDMS	1.1 ± 0.05
PSF-5PDMS	1.7 ± 0.06
PSF-1PEBAX	0.3 ± 0.10
PSF-3PEBAX	0.8 ± 0.09
PSF-5PEBAX	1.2 ± 0.14

**Table 4:** EDX surface mapping result for the as spun hollow fiber membranes.

Membrane	Trace Element (at %)				
	Carbon (C)	Oxygen (O)	Sulphur (S)	Silicon (Si)	Nitrogen (N)
PSF	79.84	17.88	2.28	Not detected	Not detected
PSF-1PDMS	79.67	15.41	3.32	1.60	Not detected
PSF-3PDMS	78.60	15.71	2.97	2.72	Not detected
PSF-5PDMS	70.57	23.14	1.55	4.74	Not detected
PSF-1PEBAX	79.66	15.34	3.44	Not detected	1.56
PSF-3PEBAX	78.83	15.60	2.98	Not detected	2.59
PSF-5PEBAX	71.95	21.27	1.80	Not detected	4.98

**Table 5:** Effect of PDMS and PEBAX coating on the PSF membrane with respect to gas selectivity.

Membrane	Selectivity (α)
PSF	4.13 ± 0.14
PSF-1PDMS	4.20 ± 0.23
PSF-3PDMS	4.56 ± 0.15
PSF-5PDMS	4.17 ± 0.12
PSF-1PEBAX	3.94 ± 0.09
PSF-3PEBAX	3.62 ± 0.10
PSF-5PEBAX	3.60 ± 0.12

**Table 6:** Comparison of the surface-coated hollow fiber membrane for oxygen/nitrogen separation.

Membrane	Permeance (GPU)		Permeability (Barrer)		αO_2_/N_2_	Reference
	O_2_	N_2_	O_2_	N_2_		
Pristine PEBAX 1567	–	–	3.30	1.30	2.54	Bernado et al. [33]
PEG-POSS with 10 wt % PEBAX 1567	–	–	4.29	1.43	3.00	Rahman et al. [34]
PEG-POSS with 20 wt % PEBAX 1567	–	–	4.57	1.86	2.46	Rahman et al. [34]
PEG-POSS with 30 wt % PEBAX 1567	–	–	7.14	2.85	2.51	Rahman et al. [34]
TFC-RO membrane with PDMS	21.65	46.76	4.54	2.10	2.16	Moradi et al. [35]
^a^ PSF with 5 wt % PDMS coating	11.00	21.01	–	–	1.91	Prajapati et al. [32]
^a^ PSF with 10 wt % PDMS coating	3.40	5.95	–	–	1.75	Prajapati et al. [32]
^a^ PSF with 15 wt % PDMS coating	2.75	10.75	–	–	3.91	Prajapati et al. [32]
^a^ PSF with 20 wt % PDMS coating	2.16	8.27	–	–	3.83	Prajapati et al. [32]
^b^ PSF + 5 wt % CXb ^c^	17.8	95.41	13.40	2.50	5.36	Magueijo et al. [12]
^b^ PSF + 10 wt % CX	16.5	78.71	12.40	2.60	4.77	Magueijo et al. [12]
^b^ PSF + 5 wt % μCX ^d^	15.3	110	11.50	1.60	7.19	Magueijo et al. [12]
PSF-3PDMS (3 wt % PDMS)	73.25	16.05	18.31	4.01	4.56	In this work
PSF-1PEBAX (1 wt % PEBAX)	48.91	12.42	12.23	3.11	3.94	In this work

^a^ Hollow fiber membrane made of 22 wt % PSF, 39 wt % DMAc, and 39 wt % THF; ^b^ Hollow fiber membrane made of 22 wt % PSF, 32 wt % DMAc, 32 wt % THF, and 14% EtOH; ^c^ Cx is the abbreviation for aerogel; ^d^ μCx is the abbreviation for microaerogel.

## Supplementary Materials

The following are available online at http://www.mdpi.com/2073-4360/10/2/126/s1. Figure S1: Cross sectional SEM image of PSF membrane coated with PDMS concentration of (a) 1 wt % (b) 3 wt % and (c) 5 wt %; Figure S2: Cross sectional SEM image of PSF membrane coated with PEBAX at concentration of (a) 1 wt % (b) 3 wt % and (c) 5 wt %; Figure S3: EDX surface marking for PSF membrane coated with PDMS and PEBAX.
