# Supplementary material for: Preparation, Characterization, and Performance Evaluation of Polysulfone Hollow Fiber Membrane with PEBAX or PDMS Coating for Oxygen Enhancement Process
Source: Polymers (Basel). 2018 Jan 28;10(2):126. doi: 10.3390/polym10020126 (PMC6414963; doi:10.3390/polym10020126)
Supplement: Supplementary file 1 [file polymers-10-00126-s001.pdf]

Preparation, Characterization and Performance Evaluation of Polysulfone Hollow Fiber Membrane with PEBAX or PDMS Coating for Oxygen Enhancement Process

Kok Chung Chong [1,*], Soon Onn Lai [1], Woei Jye Lau [2,*], Hui San Thiam [1], Ahmad Fauzi Ismail [2] and Rosyiela Azwa Roslan [2]

1. Lee Kong Chian Faculty of Engineering and Science, Universiti Tunku Abdul Rahman, Jalan Sungai Long, Bandar Sungai Long, 43300 Kajang, Malaysia; laiso@utar.edu.my (S.O.L.); thiamhs@utar.edu.my (H.S.T.)
2. Advanced Membrane Technology Research Centre (AMTEC), Universiti Teknologi Malaysia, 81310 Skudai, Johor, Malaysia; afauzi@utm.my (A.F.M.); rosyielaazwa.roslan@yahoo.com (R.A.R.)

* Correspondence: chongkc@utar.edu.my (K.C.C.); lwoeijye@utm.my (W.J.L.); Tel.: +60-3-9086-0288(K.C.C.); +60-7-553-6122(W.J.L.)

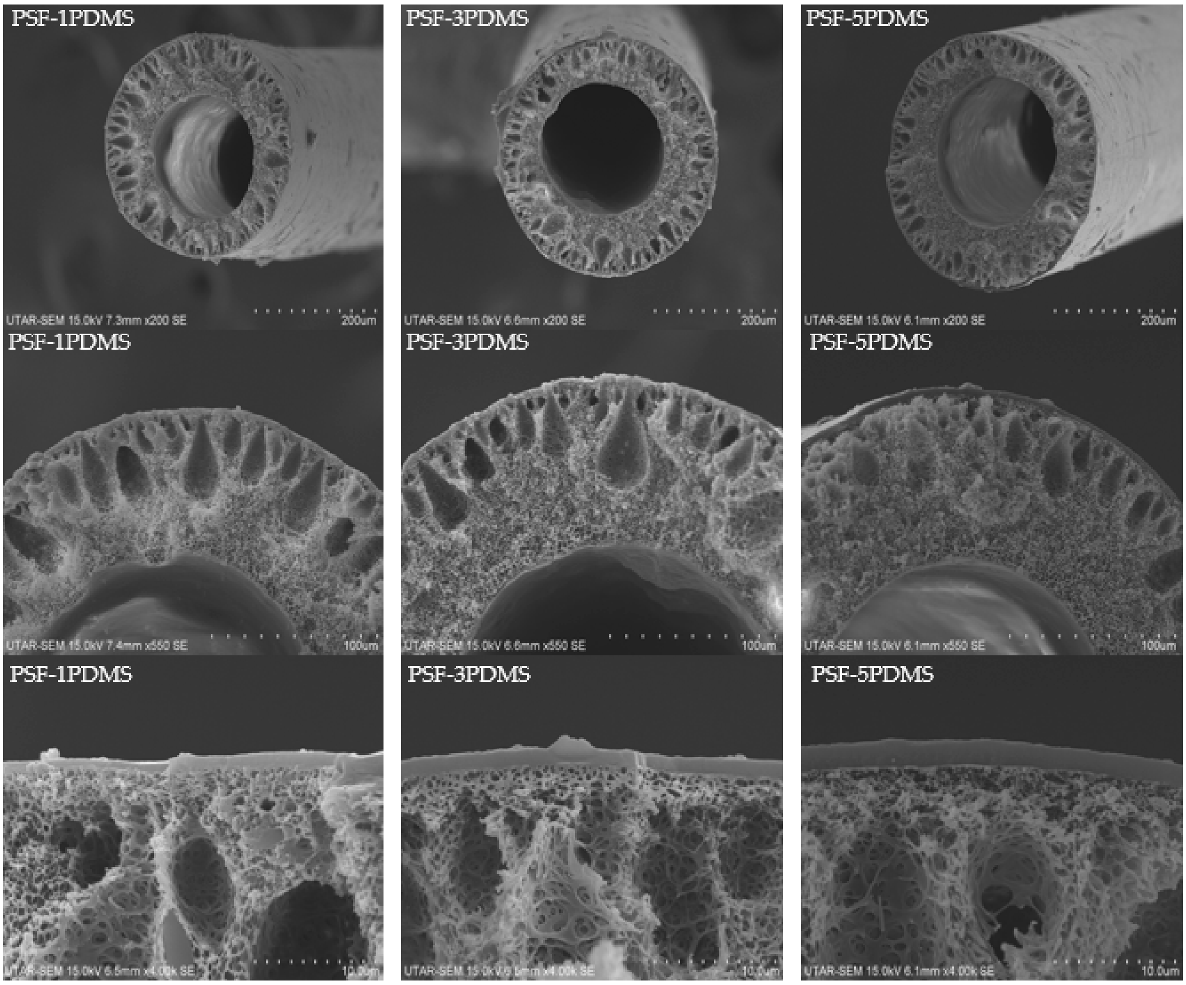

(a) (b) (c)

Figure S1. Cross sectional SEM image of PSF membrane coated with PDMS concentration of (a) 1 wt %, (b) 3 wt % and (c) 5 wt %.

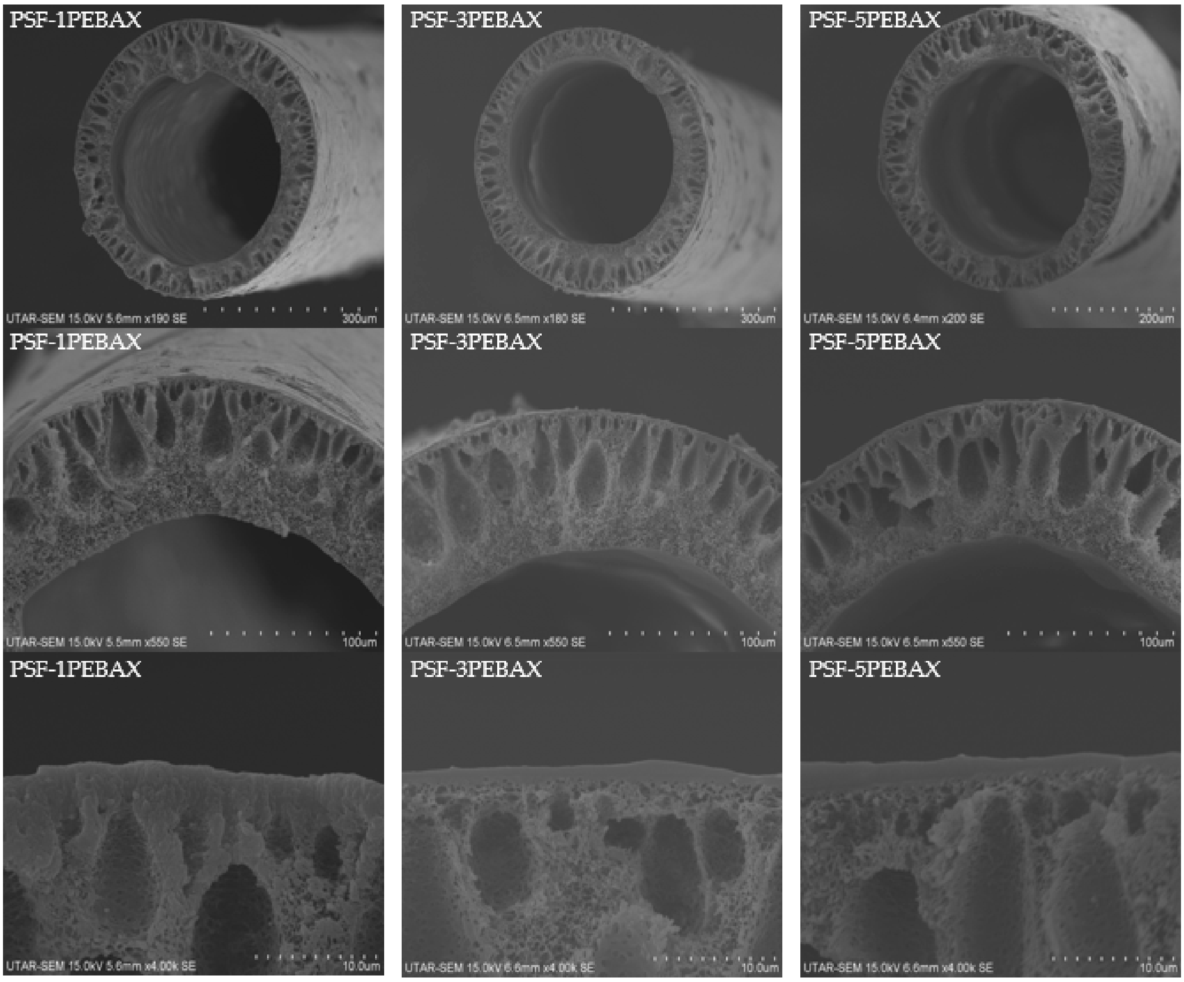

(a) (b) (c)

Figure S2. Cross sectional SEM image of PSF membrane coated with PEBAX at concentration of (a) 1 wt %, (b) 3 wt % and (c) 5 wt %.

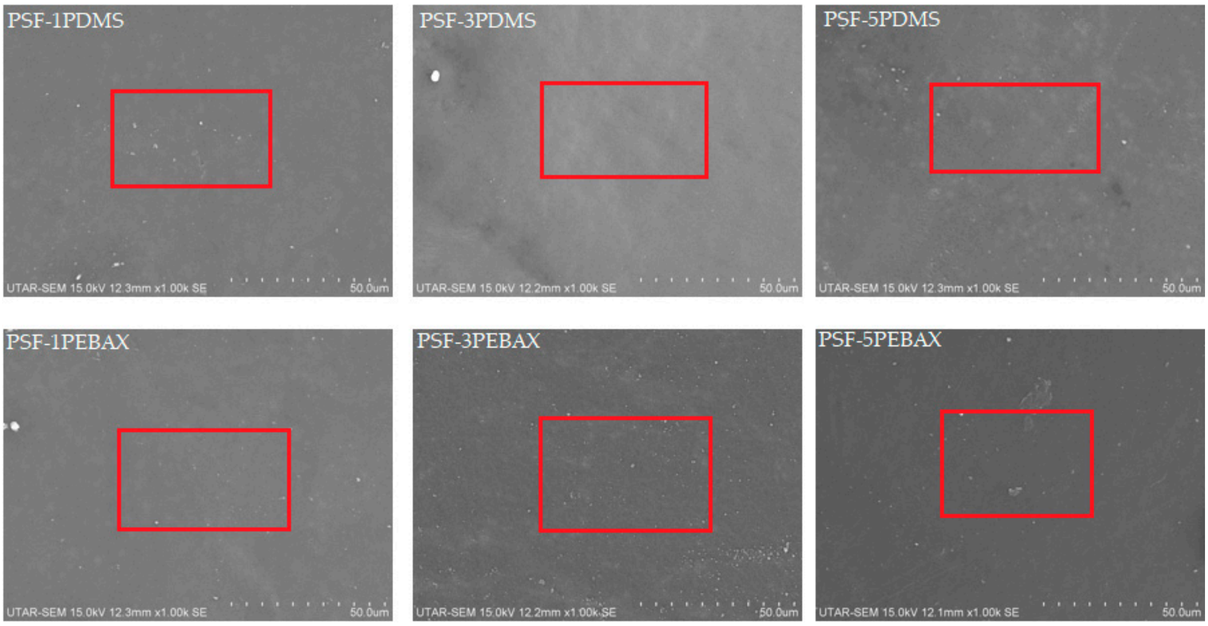

Figure S3.EDX surface marking for PSF membrane coated with PDMS and PEBAX.
